# Constructing Smaller Pauli Twirling Sets for Arbitrary Error Channels

**DOI:** 10.1038/s41598-019-46722-7

**Published:** 2019-08-02

**Authors:** Zhenyu Cai, Simon C. Benjamin

**Affiliations:** Department of Materials, University of Oxford, Oxford, UK

**Keywords:** Quantum information, Quantum simulation

## Abstract

Twirling is a technique widely used for converting arbitrary noise channels into Pauli channels in error threshold estimations of quantum error correction codes. It is vitally useful both in real experiments and in classical quantum simulations. Minimising the size of the twirling gate set increases the efficiency of simulations and in experiments it might reduce both the number of runs required and the circuit depth (and hence the error burden). Conventional twirling uses the full set of Pauli gates as the set of twirling gates. This article provides a theoretical background for Pauli twirling and a way to construct a twirling gate set with a number of members comparable to the size of the Pauli basis of the given error channel, which is usually much smaller than the full set of Pauli gates. We also show that twirling is equivalent to stabiliser measurements with discarded measurement results, which enables us to further reduce the size of the twirling gate set.

## Introduction

Twirling is a technique that has been long established in the quantum information literature. It was first used for mapping a diverse range of states into a canonical form in entanglement purification^1,2^. Then it appeared again as an integral part in randomised benchmarking^3,4^ and was also used to reduce the number of experimental runs needed in quantum process tomography^5,6^, both are critical in benchmarking the performance of quantum systems, especially “Noisy Intermediate-Scale Quantum” (NISQ) systems^7^. More recently, twirling was used as means to boost the performance of NISQ through error mitigations^8–11^ in which it enables a controlled increase of the gate error rates for error extrapolations. In this article, twirling is discussed as a technique for simulating noise and the impact of the noise on the performance of quantum error correction codes^12^.

The Gottesman-Knill theorem^13,14^ states that any quantum circuits involving only Clifford gates can be perfectly simulated in polynomial time on a classical computer. One important example is the circuits used to implement quantum error correction codes. For each code, there exists an error threshold of the circuit components below which the computational error can be made arbitrarily small by scaling up the code. As we try to obtain the error thresholds of the codes, we often need to introduce various forms of noise into the circuits based on the underlying physical implementations. This noise can be viewed as extra probabilistic gates on top of the perfect Clifford gates. However, the fact that this noise can be non-Clifford means that the circuits cannot be simulated efficiently classically, i.e. numerically determining the threshold becomes intractable.

This can be solved by twirling. Twirling means that every time we run the circuit, we conjugate the noise with an gate randomly chosen from a set of gates called the twirling set. By choosing the twirling set to be the full set of Pauli operators, we can convert any noise channel into a Pauli channel whose noise elements correspond to the Pauli basis of the original noise^15^. Such Pauli channel approximation has been shown to be effective in logical error estimation and error threshold estimation^12,16,17^, which justify its usage in error threshold simulation across various architectures^18–21^. There have also been investigations into the difference between Pauli noise channel and general noise channel in the context of entanglement distillation by Miyake *et al*.^22^.

In this article we will focus on Pauli twirling, whose twirling set is a subset of Pauli gates, with the goal of converting a given noise channel into a Pauli channel. For such a goal, twirling over the full set of Pauli operators is not always optimal. If we want to apply twirling in quantum simulations or real experiments, a twirling set with a smaller size means a lower number of simulations or experiments may be needed to get the full statistical result. Moreover, a smaller twirling set allows us to choose twirling gates that have higher fidelities and/or act on fewer qubits. This will reduce the number of errors we introduce into the system due to twirling.

In this article, we will introduce a way to exploit the symmetries in the noise channel to reduce the size of the Pauli twirling set needed for the channel. The paper is organised as follows. In Section 2, we first introduce some essential concepts for our analysis. In Section 3, we introduce the theory of Pauli twirling, in which we obtain the requirement on the twirling set. In Section 4, we show a way to construct a twirling set that satisfied the conditions that we laid out. This is followed by two examples. In Section 5, we discuss how to use stabiliser measurements to further reduce the size of our twirling set. Lastly, Section 6 provides a summary of our results and some possible future directions. The mathematical justification for our method of construction of the twirling set is described in the appendices, which forms an essential part of the paper.

## Definitions of Functions and Operations

### The pauli operator set and the * operation

*G* is defined to be the set of *n*-qubit Pauli operators:

1 $$G={\{I,X,Y,Z\}}^{\otimes n}$$

For the Pauli operator set *G*, we can define a composition rule *, which is the same as the usual Pauli matrix multiplication but ignoring all the ±1 and ±*i* factors. For one qubit we have:

$$\begin{array}{rcl}X\ast X & = & Y\ast Y=Z\ast Z=I\\ Z\ast Y & = & Y\ast Z=X\\ Z\ast X & = & X\ast Z=Y\\ Y\ast X & = & X\ast Y=Z\end{array}$$

and any composition with the identity *I* will just return the same operator.

The *n*-qubit case is just the tensor product of the one-qubit case. Note that * is commutative.

### Commutator function ***ζ***

For *g*_*i*_, *g*_*j*_ ∈ *G*, their commutator function *ζ*(*g*_*i*_, *g*_*j*_) is defined to be:

$${g}_{i}{g}_{j}=\zeta ({g}_{i},{g}_{j}){g}_{j}{g}_{i}$$

i.e.

$$\zeta ({g}_{i},{g}_{j})=(\begin{array}{ll}1\, & {\rm{for}}\,[{g}_{i},{g}_{j}]=0\\ -1\, & {\rm{for}}\{{g}_{i},{g}_{j}\}=0\end{array}$$

It follows that (see Appendix B)

2 $$\begin{array}{rcl}\zeta ({g}_{i}\,\ast \,{g}_{j},{g}_{k}) & = & \zeta ({g}_{i}{g}_{j},{g}_{k})=\zeta ({g}_{i},{g}_{k})\zeta ({g}_{j},{g}_{k})\\ \zeta ({g}_{k},{g}_{i}\,\ast \,{g}_{j}) & = & \zeta ({g}_{k},{g}_{i}{g}_{j})=\zeta ({g}_{k},{g}_{i})\zeta ({g}_{k},{g}_{j})\end{array}$$

## Twirling

### Super-operators and error channels

We use  to denote a super-operator:

A general error channel ${\mathcal E}$ is of the form:

In the following sections we are going to focus on only one of the noise operators *M*.

### Exact twirling and random twirling

One can think of twirling as a super-super-operator that turns one super-operator into another. Applying exact twirling ${{\mathcal{t}}}_{W}$ using the twirling set *W* on the noise operator *M* is defined as:3

In other words, each time we run the circuit, we conjugate the noise operator *M* with a different twirling gate *w* from the twirling set *W*. After we iterate over the whole twirling set *W* and take the average of the results, we effectively have process above.

The goal of twirling is to turn the noise operator *M* into a Pauli channel:where *p*_*g*_ is the probability of the Pauli error *g* happening, which can be 0.

On the other hand, in random twirling, instead of systematically iterating over the whole twirling set *W*, each run we choose a random element *w*_*n*_ from the twirling set *W*:

At finite *N*, there will be shot noise associated with the output of random twirling due to imperfect sampling over the twirling set. The shot noise can be reduced by increasing the number of runs *N*, allowing the effect of random twirling to approach the effect of exact twirling:

$$\mathop{\mathrm{lim}}\limits_{N\to \infty }{{\mathcal{t}}}_{W,N}^{rand}={{\mathcal{t}}}_{W}.$$

In this paper, we will focus on exact twirling, but most of the results are also applicable to random twirling.

### One-gate twirling

Let us consider the special case where *W* = {*I*, *w*}, for which *W* only contains one extra gate other than the identity.

We will call this a one-gate twirling operation and denote it as ${{\mathcal{t}}}_{\{I,w\}}$.

Doing nested one-gate twirling with ${{\mathcal{t}}}_{\{I,{w}_{1}\}}$ on top of ${{\mathcal{t}}}_{\{I,{w}_{2}\}}$ on top of ${{\mathcal{t}}}_{\{I,{w}_{3}\}}$, etc, is equivalent to twirling with *W* = *w*_1_, *w*_2_, …, where *w*_1_, *w*_2_, … denotes the full set of gates that can be generated from {*w*_1_, *w*_2_, …} using operation *.

$${{\mathcal{t}}}_{\{I,{w}_{1}\}}\cdot {{\mathcal{t}}}_{\{I,{w}_{2}\}}\cdots ={{\mathcal{t}}}_{\langle {w}_{1},{w}_{2},\cdots \rangle }$$

### Requirements and results of twirling

Now we will focus on Pauli twirling, which means our twirling set consists of only Pauli operators: *W*⊆*G*. Note that all Pauli operators are Hermitian: $w={w}^{\dagger }$.

We can break any *n*-qubit noise operator *M* into its Pauli basis:

$$\begin{array}{rcl}M & = & \frac{1}{{2}^{n}}\sum \,_{g\in G}{\rm{Tr}}(gM)g\\  & = & \frac{1}{{2}^{n}}\sum _{v\in V}\,{\rm{Tr}}(vM)v\end{array}$$

where *V* is the Pauli basis of *M*:

$$V=\{g\in G|{\rm{Tr}}(gM)\ne 0\}$$

Substituting this into (3) and applying it onto a state *ρ*, we have:4

Now let us look at sum over *W*. Using (2), we have

5 $$\begin{array}{c}\sum _{w\in W}\,wvw\rho wv^{\prime} w\\ \,=v\rho v^{\prime} \sum _{w\in W}\,\zeta (w,v)\zeta (w,v^{\prime} )\\ \,=v\rho v^{\prime} \sum _{w\in W}\,\zeta (w,vv^{\prime} )\end{array}$$

Substituting this into (4) we get:6where we have made use the fact that $\zeta (w,vv)=\zeta (w,I)=1$.

To construct a Pauli noise channel, we want the *v* ≠ *v*′ term to vanish (see Appendix C where we show that this is a necessary condition). This can be achieved by choosing a *W* such that

7 $$\sum _{w\in W}\,\zeta (w,vv^{\prime} )=0\,\forall v,v^{\prime} \in V\,{\rm{and}}\,v\ne v^{\prime} $$

In such a case, the result of twirling the noise operator *M* is just8

Our arguments can be easily extended to the full noise channel (Section 3.1) by adding $\sum _{M}$ before all the equations. In such case, *V* will be re-defined as the Pauli basis needed to construct all the noise elements in the noise channel. All the other results follow.

The details of how to apply twirling on erroneous quantum components and the result of such twirling is outlined in Appendix A.

## Construction of the Twirling Set

As we can see from the last section, the key to twirling is to find a twirling set *W* that satisfy (7) for the Pauli basis *V* of the given noise.

The common choice is *W* = *G*, the full set of Pauli operators. In such a way, for any *v* ≠ *v*′ (i.e. *vv*′ ≠ *I*), the number of elements in *G* that commute with *vv*′ will always equal to the number of elements that anti-commute with *vv*′, thus (7) is always satisfied.

Hence, if we choose *W* = *G*, we can transform any error channel into a Pauli channel.

However, as mentioned before, twirling with the full Pauli set is not always ideal. A systematic way to construct a smaller set of *W* is laid out in this section, whose validity is proven in Appendix D, E, ??. Note that for the steps below, compositions between elements refer to the * operation defined in Section 2.1.

Before proceeding to the steps of construction, we need to introduce the ideas of commutator table first which is crucial to our method of construction.

### Commutator table

#### Definition

For *A* ⊆ *G*, *B* ⊆ *G*, a commutator table *ζ*(*a*_*i*_, *b*_*j*_) is defined to be

Following (2), we then have

9 $$\begin{array}{rcl}{\rm{row}}\,{\rm{composition}}:\zeta (a,{b}_{j})\zeta (a^{\prime} ,{b}_{j}) & = & \zeta (a\,\ast \,a^{\prime} ,{b}_{j})\\ {\rm{column}}\,{\rm{composition}}:\zeta ({a}_{i},b)\zeta ({a}_{i},b^{\prime} ) & = & \zeta ({a}_{i},b\,\ast \,b^{\prime} )\end{array}$$

#### Generator table $\zeta ({\tilde{q}}_{i},{\tilde{h}}_{j})$

Generator tables are just commutator tables of the form:

$$\zeta ({\tilde{q}}_{i},{\tilde{h}}_{j})=1-2{\delta }_{ij}$$

Example generator tables for different sizes of $\tilde{H}$ are shown in Table 1.

**Table 1:**
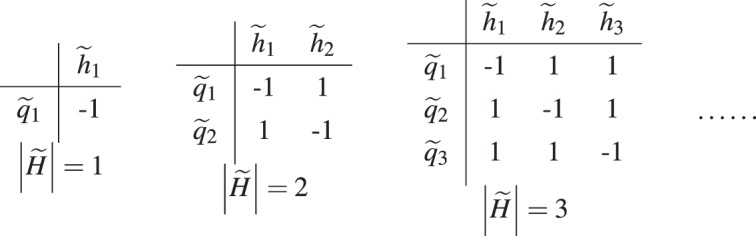
Generator tables $\zeta ({\tilde{q}}_{i},{\tilde{h}}_{j})$ for different $\tilde{H}$.

Note that by definition, we have

10 $$|\tilde{H}|=|\tilde{Q}|$$

The rows of a generator table cannot be obtained from composing other rows, thus the row labels ${\tilde{q}}_{i}$ also cannot be obtained from composing other row labels. Hence, all the row labels ${\tilde{q}}_{i}$ are independent from each other, forming a valid generating set. Similarly for the column labels ${\tilde{h}}_{j}$, hence the name generator tables.

We can compose the columns of the generator table to obtain new columns as shown in Table 2.

**Table 2:**
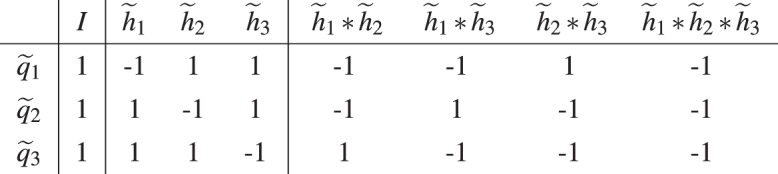
One of the commutator tables $\zeta ({\tilde{q}}_{i},{h}_{s,j})$ obtained by composing the columns of the generator table of size $|\tilde{H}|=3$ in Table 1. Here ${h}_{s,j}\in {H}_{S}\subseteq H=\langle \tilde{H}\rangle$.

### Steps to construct *W*

1. Decompose the noise operator *M* to obtain its Pauli basis *V*
  $$V=\{g\in G|{\rm{Tr}}(gM)\ne 0\}$$
  For a general noise channel, *V* will be the union of the Pauli basis of all the noise elements in the noise channel.
2. Find the following set:
  $\tilde{V}$: the generating set of $V$.
  ${\tilde{V}}_{S}$: the subset of elements in $\tilde{V}$ that are used to generate elements in $V-\tilde{V}$.
3. Find the smallest integer *N* that satisfies both
  $$\begin{array}{rcl}N & \ge  & {\mathrm{log}}_{2}(|V|)\\ N & \ge  & |{\tilde{V}}_{S}|\end{array}$$
  We now define a generating set $\tilde{H}$ of size *N* and denote the complete set that it generates as $H=\langle \tilde{H}\rangle$
4. Map elements in *V* to elements in *H* using the following steps:
  (a) Map ${\tilde{V}}_{S}$ to a subset of elements in $\tilde{H}$
  (b) Map the elements in $V-\tilde{V}$ to elements in $H-\tilde{H}$ by following the composition relations of the elements in ${\tilde{V}}_{S}$.
  (c) Map elements in $\tilde{V}-{\tilde{V}}_{S}$ to any subset of the remaining elements in *H* (which includes the identity).
  Using the steps above, we can obtain the subset of *H* that $\tilde{V}$ maps to, which we will denoted as ${H}_{\tilde{V}}$:
  $${\tilde{v}}_{i}\mapsto {h}_{\tilde{v},i}\,{\rm{for}}\,{\tilde{v}}_{i}\in \tilde{V}\,{\rm{and}}\,{h}_{\tilde{v},i}\in {H}_{\tilde{V}}$$
5. Starting with the generator table $\zeta ({\tilde{q}}_{i},{\tilde{h}}_{j})$ of size $|\tilde{H}|$, we compose its columns to get the commutator table $\zeta ({\tilde{q}}_{i},{h}_{\tilde{v},j})$ (See Section 4.1.2).
6. The twirling generating set $\tilde{W}$ is constructed such that $\zeta ({\tilde{w}}_{i},{\tilde{v}}_{j})=\zeta ({\tilde{q}}_{i},{h}_{\tilde{v},j})$ for all *i* and *j*.
7. After finding $\tilde{W}$, we can opt to twirl the error by doing nested one-gate twirling (Section 3.3) using the elements in $\tilde{W}$. Or equivalently, we can twirl the error using the full set of $W=\langle \tilde{W}\rangle$

Note that $\tilde{W}$ is not unique because the generating sets are not unique.

### An example

Here we will ignore the qubit labels on the operators. e.g. *IX* ≡ *I*_1_*X*_2_.

1. Suppose we have noise
  $$M\propto IX+IZ+YX+\frac{1}{\sqrt{2}}ZX+YY$$
  then the Pauli basis of *M* is
  $$V=\{IX,IZ,YX,ZX,YY\}$$
2. Within *V*, the only composition relation is *YY* = *IZ* * *YX*. Hence, we have:
  $$\begin{array}{rcl}{\tilde{V}}_{S} & = & \{IZ,YX\}\\ \tilde{V} & = & \{IX,\,IZ,\,YX,\,ZX\}\end{array}$$
3. The smallest integer *N* that satisfies both
  $$\begin{array}{rcl}N & \ge  & {\mathrm{log}}_{2}(|V|)=2.58\\ N & \ge  & {|\tilde{V}|}_{S}=2\end{array}$$
  is *N* = 3. Hence, we will define a generating set $\tilde{H}$ of size 3.
4. Find the mapping $\tilde{V}\mapsto {H}_{\tilde{V}}\subseteq H=\langle \tilde{H}\rangle$:

- Map ${\tilde{V}}_{S}$ to a subset of elements in $\tilde{H}$
  $${\tilde{V}}_{S}=\{IZ,YX\}\mapsto \{{\tilde{h}}_{1},{\tilde{h}}_{2}\}\subseteq \tilde{H}$$
- Map elements in $V-\tilde{V}$ to elements in $H-\tilde{H}$ by following the way we use ${\tilde{V}}_{S}$ to generate elements in $V-\tilde{V}$:
  $$V-\tilde{V}=\{YY=IZ-YX\}\mapsto \{{\tilde{h}}_{1}\ast {\tilde{h}}_{2}\}$$
- Map $\tilde{V}-{\tilde{V}}_{S}$ to any subset of the remaining elements in *H*:

$$\tilde{V}-{\tilde{V}}_{S}=\{IX,ZX\}\mapsto \{I,{\tilde{h}}_{3}\}$$

Hence, we find:

$$\tilde{V}=\{IX,IZ,YX,ZX\}\mapsto {H}_{\tilde{V}}=\{I,{\tilde{h}}_{1},{\tilde{h}}_{2},{\tilde{h}}_{3}\}$$

5. Starting with the generator table of $|\tilde{H}|=3$, we can construct the commutator table $\zeta ({\tilde{q}}_{i},{h}_{\tilde{v},j})$:
  In the brackets are the elements in $\tilde{V}$ that the elements in ${H}_{\tilde{V}}$ map to.
6. Our goal is just to find $\tilde{W}$ such that $\zeta ({\tilde{w}}_{i},{\tilde{v}}_{j})=\zeta ({\tilde{q}}_{i},{h}_{\tilde{v},j})$.
  A possible choice is $\tilde{W}=\{IX,ZI,YI\}$, which produces the following commutator table
  This is the same as the commutator table in the last step.
7. Twirling of *M* can be achieved using nested one-gate twirling over the elements in $\tilde{W}$:

$${{\mathcal{t}}}_{\{I,IX\}}\cdot {{\mathcal{t}}}_{\{I,ZI\}}\cdot {{\mathcal{t}}}_{\{I,YI\}}$$

Or equivalently, we can twirl over the full twirling set of

$$\begin{array}{rcl}W & = & \langle \tilde{W}\rangle \\  & = & \{II,\,IX,\,ZI,\,YI,\,ZX,\,YX,\,XI,\,XX\}\end{array}$$

Using (8), the result of twirling the noise operator *M* is just

### Application to a physical noise model

**Figure 1:**
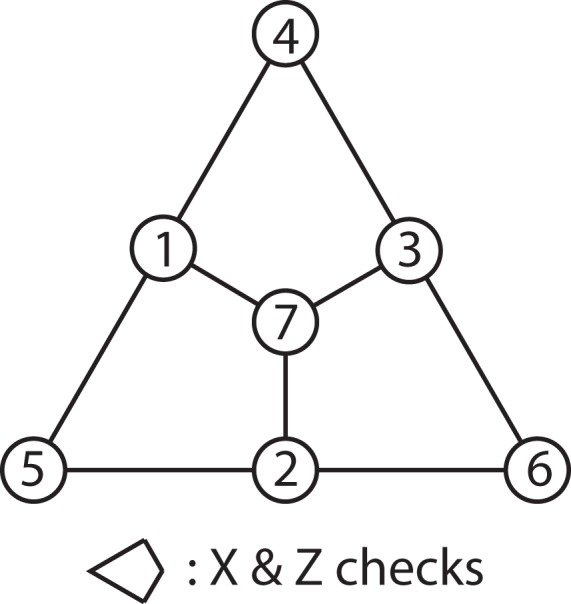
The Structure of Steane code.

We will provide another example that has more physical significance. Suppose we want to implement the Steane code as shown in Fig. 1 using spin qubits, but there is a small global field causing a global rotation of a small angle *θ* in the *Z* direction, leading to the following coherent noise:

$$M=\exp \{-i\theta \sum _{i=1}^{7}\,{Z}_{i}\}=I-i\theta \sum _{i=1}^{7}\,{Z}_{i}+O({\theta }^{2})$$

We will ignore the higher order term *O*(*θ*^2^) in the noise channel for the purpose of obtaining the reduced twirling set. The exact steps of obtaining the reduced twirling set are:

1. The Pauli basis of *M* is
  $$V=\{I\}+\{{Z}_{n}|n\in {\mathbb{N}},1\le n\le 7\}$$
2. Within *V*, there is no composition relations other than those involving the identity. Hence, we have:
  $$\begin{array}{rcl}\tilde{V} & = & \{{Z}_{n}|n\in {\mathbb{N}},1\le n\le 7\}\\ {\tilde{V}}_{S} & = & \{{Z}_{1}\}\end{array}$$
3. The smallest integer *N* that satisfies both
  $$\begin{array}{rcl}N & \ge  & {\mathrm{log}}_{2}(|V|)=3\\ N & \ge  & |{\tilde{V}}_{S}|=1\end{array}$$
  is *N* = 3. Hence, we will define a generating set $\tilde{H}$ of size 3.
4. Using the fact that ${\tilde{V}}_{S}=\{{Z}_{1}\}$, the following mapping $\tilde{V}\mapsto {H}_{\tilde{V}}\subseteq H=\langle \tilde{H}\rangle$ can be found:
  $$\{{Z}_{1},{Z}_{2},{Z}_{3},{Z}_{4},{Z}_{5},{Z}_{6},{Z}_{7}\}\mapsto $$
  $$\{{\tilde{h}}_{1},\,{\tilde{h}}_{2},\,{\tilde{h}}_{3},\,{\tilde{h}}_{1}\,\ast \,{\tilde{h}}_{2},\,{\tilde{h}}_{1}\,\ast \,{\tilde{h}}_{3},\,{\tilde{h}}_{2}\ast {\tilde{h}}_{3},\,{\tilde{h}}_{1}\,\ast \,{\tilde{h}}_{2}\,\ast \,{\tilde{h}}_{3}\}$$
5. Now starting with the generator table of $|\tilde{H}|=3$, we can construct the commutator table $\zeta ({\tilde{q}}_{i},{h}_{\tilde{v},j})$:
  In the brackets are the elements in $\tilde{V}$ that the elements in ${H}_{\tilde{V}}$ map to.
6. Our goal is just to find $\tilde{W}$ such that $\zeta ({\tilde{w}}_{i},{\tilde{v}}_{j})=\zeta ({\tilde{q}}_{i},{h}_{\tilde{v},j})$. A possible choice is to have $\tilde{W}=\{{X}_{1}{X}_{4}{X}_{5}{X}_{7},\,{X}_{2}{X}_{4}{X}_{6}{X}_{7},\,{X}_{3}{X}_{5}{X}_{6}{X}_{7}\}$, which will produce the following commutator table:

This is the same as the commutator table in the last step.

**Figure 2:**
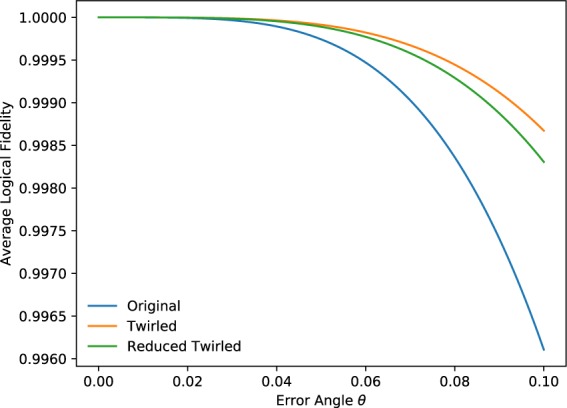
The logical fidelity of Steane code under coherent global Z noise.

The result of applying this reduced twirling generating set (*X*_1_*X*_4_*X*_5_*X*_7_, *X*_2_*X*_4_*X*_6_*X*_7_, *X*_3_*X*_5_*X*_6_*X*_7_) as opposed to the full twirling set to the Steane code under global Z rotation is shown in Fig. 2. As we can see from the plot, the fidelity curve of the reduced twirling set has some deviation from the full twirling curve due to the approximation we made initially in which we discarded the *O*(*θ*^2^) term. However, when looking at *θ* = 0.1, such deviation is ~3.5 × 10^−4^, which is an order of magnitude smaller than the ~2.5 × 10^−3^ deviation of the untwirled curve from the twirled curve. If we assume that when we sample from the full twirling set, the fluctuation of the logical fidelity in each sample is represented by the gap between the twirled fidelity and the original fidelity (since the original curve is just twirling using identity), then to achieve the same level of approximation as our reduced twirling set, we need to reduce the error fluctuation by an order of magnitude, which requires 10^2^ = 100 samples (i.e. 100 circuit runs). This is significantly more than the 2^3^ = 8 circuit runs needed using the reduced twirling set.

### Expected size of $\tilde{W}$

Using (S11), (S12), (S13) and $|W|={2}^{|\tilde{W}|}$, we have

$${\mathrm{log}}_{2}(|V|)\le |\tilde{W}|\le |\tilde{V}|$$

$$|V|\le |W|\le {2}^{|\tilde{V}|}$$

Hence, unlike the full Pauli operator set whose size 4^*n*^ is dependent on the number of qubits *n* that we are considering, the size of our twirling set |*W*| is only dependent on the sizes of the Pauli basis and the generating set of the Pauli basis of the particular noise channel we have. Noise arising from real physical process usually have symmetries present. Such symmetry constraints will reduce the size of the Pauli basis that builds our noise, which enable us to find a much smaller twirling set than the full Pauli set.

One such example was shown in the last section (Section 4.4), in which the lower bound is reached: $|\tilde{W}|={\mathrm{log}}_{2}(|V|)$. For such noise due to the fluctuation of a global field, we have |*V*| = *n* + 1 where *n* is the number of qubits. Hence, we have $|\tilde{W}|={\mathrm{log}}_{2}(n+1)$. Comparing to the twirling using the full set of Pauli operators: $|\tilde{W}|=2n$, there is an exponential reduction of the size of the twirling set.

## Twirling and Measurements in Stabiliser Code

### Stabiliser code

In quantum error correction codes, we try to encode logical qubits into a larger number of physical qubits. All the states of the logical qubits $|{\psi }_{L}\rangle$ will live in a subspace ${{\mathcal{V}}}_{{\mathcal{t}}}$ of the full quantum space of the physical qubits. We will call ${{\mathcal{V}}}_{{\mathcal{t}}}$ the code subspace. Quantum states that live outside the code subspace can be detected as erroneous states and might be corrected by projecting (or transforming) back to the code subspace.

If we have a given code subspace ${{\mathcal{V}}}_{{\mathcal{t}}}$. Then the stabiliser set *S* ⊆ *G* is defined to be:

$$S=\{s\in G|s|{\psi }_{L}\rangle =|{\psi }_{L}\rangle \,\forall |{\psi }_{L}\rangle \in {{\mathcal{V}}}_{{\mathcal{t}}}\}$$

Hence, for any $s\in S$ we have

$$\begin{array}{rcl}(\frac{1+s}{2})|{\psi }_{L}\rangle  & = & |{\psi }_{L}\rangle \\ (\frac{1-s}{2})|{\psi }_{L}\rangle  & = & 0\end{array}$$

### Equivalence of one-gate twirling and stabiliser checks

#### One-gate twirling

For a given noise operator *M* and a given one-gate twirling set *W* = {*I*, *w*}, we can write

$$M={M}_{+}+{M}_{-}$$

where *M*_+_ contains all the Pauli basis elements in *M* that commute with *w*, *M*_−_ contains all the Pauli basis elements in *M* that anti-commute with *w*.

Then using (3), we have:11i.e. an one-gate twirl *W* = {*I*, *w*} will decohere between the components in *M* that commute with *w* and the components that anti-commute with *w*.

#### Stabiliser checks

For a given noise operator *M* and a given stabiliser *s*, we can write

$$M={M}_{+}+{M}_{-}$$

where *M*_+_ contains all the Pauli basis elements in *M* that commute with *s*, *M*_−_ contains all the Pauli basis elements in *M* that anti-commute with *s*.

Then if such noise happens on |*ψ*_*L*_〉, and we do an *s* stabiliser check on it, then we have

$$\begin{array}{rcl}sM|{\psi }_{L}\rangle  & = & \mathop{\underbrace{\frac{1+s}{2}({M}_{+}+{M}_{-})|{\psi }_{L}\rangle }}\limits_{{\rm{projection}}\,{\rm{onto}}+1\,{\rm{state}}}+\mathop{\underbrace{\frac{1-s}{2}({M}_{+}+{M}_{-})|{\psi }_{L}\rangle }}\limits_{{\rm{projection}}\,{\rm{onto}}-1\,{\rm{state}}}\\  & = & [{M}_{+}\frac{1+s}{2}|{\psi }_{L}\rangle +{M}_{-}\frac{1-s}{2}|{\psi }_{L}\rangle ]\\  &  & +[{M}_{+}\frac{1-s}{2}|{\psi }_{L}\rangle +{M}_{-}\frac{1+s}{2}|{\psi }_{L}\rangle ]\\  & = & \mathop{\underbrace{{M}_{+}|{\psi }_{L}\rangle }}\limits_{{\rm{projection}}\,{\rm{onto}}+1\,{\rm{state}}}+\mathop{\underbrace{{M}_{-}|{\psi }_{L}\rangle }}\limits_{{\rm{projection}}\,{\rm{onto}}-1\,{\rm{state}}}\end{array}$$

Here we can see that an *s* stabiliser check that gives +1 result will collapse the state into ${M}_{+}|{\psi }_{L}\rangle$ (up to a normalising constant), an *s* stabiliser check that gives −1 result will collapse the state into ${M}_{-}|{\psi }_{L}\rangle$ (up to a normalising constant).

If we discard the information about result of the *s* stabiliser check, then our error channel after the *s* stabiliser check becomes:12where $\rho =|{\psi }_{L}\rangle \langle {\psi }_{L}|$.

We can follow similar analysis even if there is a Pauli error *g* on the logical state $|{\psi }_{L}\rangle \to g|{\psi }_{L}\rangle$. The extra Pauli error may swap the ±1 stabiliser check outcome, but will not change our error channel in (12).

Comparing (12) to (11), we have:

$${{\mathcal{t}}}_{\{I,s\}}\equiv {{\mathcal{t}}}_{s}$$

Hence, when we have a error *M* occurring on top of $g|{\psi }_{L}\rangle$, twirling with *W* = {*I*, *s*} is equivalent to performing a *s* stabiliser check and throwing away the result.

### Combining stabiliser check with twirling

As mentioned in the last step of Section 4.2, a given noise operator *M* can be twirled by doing nested one-gate twirling using the elements in the twirling generating set $\tilde{W}$. In the last section, we have shown that the *s*-base stabiliser measurement is equivalent to the one-gate twirling with *W* = {*I*, *s*}. Hence, we can use the *s* stabiliser check as a substitute for element *s* in the $\tilde{W}$ to further reduce the size of $\tilde{W}$.

This is best shown through a simple example.

Suppose we have the following circuit:Here $|{\psi }_{L}\rangle$ is stabilised by *Z*, i.e. $|{\psi }_{L}\rangle =|0\rangle$ (note that here we encode 0 logical qubits). In this circuit, we are effectively doing a *Z* stabiliser measurement on $|{\psi }_{L}\rangle$, with a noise

$$M\propto I+X+Y+Z$$

occurring in the circuit.

Now if we go through Section 4.2, we will obtain the twirling generating set $\tilde{W}$ needed to twirl *M* as follows:

$$\tilde{W}=\{X,Z\}$$

which means we need the following nested one-gate twirling circuit to turn *M* into Pauli errors:where the pair of *X* will be applied with 50% probability, similarly and independently for the pair of *Z*.

However, if we discard the information specifying the result of the *Z* stabiliser check, then as argued before, the *Z* stabiliser check will have the same effect as the *Z*-twirling. Hence, we can turn *M* into Pauli errors with just the *X*-twirling:

This example shows how stabiliser measurements with thrown-away results can lead to a smaller set of twirling gates.

## Conclusion and Future Works

In this paper, we found the necessary and sufficient conditions for a set of twirling gate to turn a given noise operator into a Pauli channel form. We then demonstrated a way to construct the smallest twirling set that satisfies the conditions. The size of the twirling set we obtained is lower-bounded by the size of the Pauli basis of the noise operator, and upper bounded by ${2}^{\tilde{V}}$ where $\tilde{V}$ is the generating set of the Pauli-basis of the noise operator. We showed that there can be an exponential reduction in the number of twirling gates in some cases. Our arguments can be easily extended to a general noise channel. In addition, we showed that in the case of stabiliser codes, we can replace elements in the generating set of the twirling set with existing stabiliser measurements to further reduce the size of the twirling set.

For twirling of a given noise operator, we have not proven the twirling set we obtained is the smallest possible. Hence, any further investigations can look into such a proof or even constructing a smaller twirling set than ours.

For a general noise channel, the simple generalisation mentioned in Section 4.2 can indeed produce a twirling set smaller than the full set of Pauli operators. However, it is not the smallest possible set since we have not made use of the fact that different noise elements are inherently separated. To obtain the optimal twirling set, we need to study the following property of twirling: if we know a twirling set that can twirl the noise operator *M*, and we know another twirling set that can twirl the noise operator *N*, then what is the twirling set that can twirl the noise channel  Similarly, we can also ask what is the twirling set that can twirl the noise operator *MN*, which is essential in finding a single twirling operation that can twirl several consecutive erroneous components. We hope that this article will provide a framework for further explorations of properties of twirling like the two mentioned above.

In this paper, we have only focused on using Pauli twirling to convert error channels into Pauli channels for error threshold estimation. There is also Clifford twirling, which converts error channels into depolarising channels instead. Clifford twirling can be viewed as symplectic twirling on top of Pauli twirling^23^, so we can easily apply our arguments to the Pauli twirling step. Clifford twirling is integral to Clifford randomised benchmarking^3,4^ and is also used in quantum process tomography to reduce the number of experiments that we need to run exponentially^5,6^. We cannot apply our techniques directly to both of these areas since we do not know the form of the quantum process that we want to twirl. However, our analysis might provide a basis for finding a reduced twirling set for the case in which some characteristics of the quantum process are known, but not the full model.

## Supplementary information

Supplementary Materials
