# Supplementary material for: Constructing Smaller Pauli Twirling Sets for Arbitrary Error Channels
Source: Sci Rep. 2019 Aug 2;9:11281. doi: 10.1038/s41598-019-46722-7 (PMC6677783; doi:10.1038/s41598-019-46722-7)
Supplement: Supplementary file 1 — Supplementary Materials [file 41598_2019_46722_MOESM1_ESM.pdf]

Constructing Smaller Pauli Twirling Sets for Arbitrary Error Channels

Zhenyu Cai[1,*] **and Simon Benjamin**[1]

[1]Department of Materials, University of Oxford, Oxford, UK
[*]zhenyu.cai@materials.ox.ac.uk

Supplemental Materials

A Twirling of Gate Noise

In real circuits, a noise operator is not a physical gate, hence it is impossible to bracket the noise operator with twirling gates. We can instead bracket the source of the noise with twirling gates..

Suppose we want to apply a gate C, but an error M occurs after gate C with a probability p:

$$\mathscr{C}_e(\rho) = (1-p)\overline{C}\rho + p\overline{MC}\rho. \tag{S1}$$

Now for the noisy part of the process $\overline{MC}\rho$, we want to twirl the noise M.

$$\begin{aligned}\mathscr{C}_e(\rho) &\xrightarrow{twirling} (1-p)\overline{C}\rho + p\mathscr{T}(\overline{M})\overline{C}\rho \\ &= (1-p)\overline{C}\rho + p\frac{1}{|W|}\sum_{w\in W}\overline{(wMw)\,C}\rho \\ &= (1-p)\overline{C}\rho + p\frac{1}{|W|}\sum_{w\in W}\overline{wMC\left(C^{\dagger}wC\right)}\rho\end{aligned} \tag{S2}$$

which is just the following circuit:

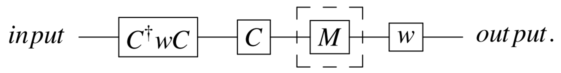

Hence, by bracketing the erroneous gate C with the twirling gate w and its complementary gate $C^{\dagger}wC$, we are effectively twirling the noise M coming out of C.

Substituting (8) into (S2) we have

$$\mathscr{C}_e(\rho) \xrightarrow{twirling} (1-p)\overline{C}\rho + \frac{p}{2^{2n}}\sum_{v\in V}|\mathrm{Tr}(vM)|^2\,\overline{vC}\rho.$$

Hence, after twirling, $\mathscr{C}_e(\rho)$ becomes a error channel with Pauli error $v \in V$ happening with the probability $\frac{p|\mathrm{Tr}(vM)|^2}{2^{2n}}$.

B Property of ζ

For $g, g' \in G$, $\zeta(g,g')$ is defined to be:

$$gg' = \zeta(g,g')g'g$$

i.e.

$$\zeta(g,g') = \begin{cases} 1 & \text{for } [g,g'] = 0 \\ -1 & \text{for } \{g,g'\} = 0 \end{cases}$$

We then have the following properties:

- $\zeta(g,g')^{-1} = \zeta(g,g')$

  Proof:

  Since $\zeta(g,g') = \pm 1$.

- $\zeta(g,g') = \zeta(g',g)$

  Proof:

  $$\begin{aligned} gg' &= \zeta(g,g')g'g \\ \zeta(g,g')^{-1}gg' &= g'g \end{aligned}$$

  Hence, $\zeta(g,g')^{-1} = \zeta(g',g)$. Then using $\zeta(g,g')^{-1} = \zeta(g,g')$, we have $\zeta(g',g) = \zeta(g,g')$.

- $\zeta(g, g_1 g_2) = \zeta(g, g_1)\zeta(g, g_2)$

  Proof:

$$\begin{aligned} g g_1 g_2 &= \zeta(g, g_1 g_2) g_1 g_2 g \\ \zeta(g, g_1) g_1 g g_2 &= \zeta(g, g_1 g_2) g_1 g_2 g \\ \zeta(g, g_1)\zeta(g, g_2) g_1 g_2 g &= \zeta(g, g_1 g_2) g_1 g_2 g \\ \zeta(g, g_1)\zeta(g, g_2) &= \zeta(g, g_1 g_2) \end{aligned}$$

- $\zeta(g, cg') = \zeta(g, g')$ for any complex number c.

  Proof:

$$\begin{aligned} gcg' &= \zeta(g, cg') cg' g \\ gg' &= \zeta(g, cg') g' g \\ \zeta(g, cg') &= \zeta(g, g') \end{aligned}$$

  From this it immediately follows that:

$$\begin{aligned} \zeta(g * g', g'') &= \zeta(cgg', g'') \\ &= \zeta(gg', g'') \end{aligned}$$

  where $*$ is the operation we defined in Section.2.1.

C Proof that (7) is a necessary condition

(6) can be written as

$$\mathscr{T}(\overline{M})\rho = \sum_{g, g' \in V} \alpha_{gg'} g \rho g'$$

If we indeed can transform this into a Pauli channel, then we have:

$$\sum_{g, g' \in G} \alpha_{gg'} g \rho g' = \sum_{g'' \in G} \beta_{g''} g'' \rho g''$$

$$\sum_{g} (\alpha_{gg} - \beta_g) g \rho g + \sum_{g \neq g', g, g' \in G} \alpha_{gg'} g \rho g' = 0$$

This should be valid for any ρ. Using the process matrix formalism, we have:

$$\sum_{g} (\alpha_{gg} - \beta_g) |g\rangle \langle g| + \sum_{g \neq g', g, g' \in G} \alpha_{gg'} |g\rangle \langle g'| = 0$$

In terms of Pauli basis states $\{|g\rangle\}$, the LHS is just a matrix with $(\alpha_{gg} - \beta_g)$ at the diagonal and $\alpha_{vv'}$ at the off diagonal. Hence, the only way for the equation to be valid is when:

$$\begin{aligned} \beta_g &= \alpha_{gg} \\ \alpha_{gg'} &= 0 \end{aligned}$$

which is equivalent to (7).

D Properties of Commutator Tables

This section proves some properties of commutator tables (See Section 4.1), which is crucial in proving our construction of twirling set is valid in Appendix E.

Note that in this section, whenever we talk about composition, we are referring to the operation $*$ (Section 2.1).

D.1 Row Commutator Group

D.1.1 Homomorphic mapping

For any $A \subseteq G$, we can construct a commutator table $\zeta(g_i, a_j)$ with $g_i \in G$. Its rows form a set R_A:

$$R_A = \{\zeta(g, a_j) \mid g \in G\}$$

From (9), we have:

$$\zeta(g * g', a_j) = \zeta(g, a_j)\zeta(g', a_j) \quad \forall g, g' \in G \tag{S3}$$

Note that The Pauli operator set G is a group under the composition rule $*$. (S3) means that there exists a homomorphic mapping: $G \mapsto R_A$.

Hence, R_A **is a also a group**.

D.1.2 Quotient sets

The kernel subgroup of the homomorphic mapping $G \mapsto R_A$ by definition is

$$K_A = \{g \in G \mid \zeta(g, a_j) = (1, 1, 1, \cdots) = \vec{1}\}$$

i.e. it maps to the set of rows in the commutator table $\zeta(g_i, a_j)$ that only contains 1.

K_A will partition G into $\frac{|G|}{|K_A|}$ cosets. For $g \in G$ that are within the same coset gK_A, their corresponding row vector $\zeta(g, a_j)$ will have the same value.

We will define the **quotient set** (not group) Q_A as a set that has one and only one element from each coset of K_A. Hence, the set of row vectors $\{\zeta(q_a, a_j) \mid q_a \in Q_A\}$ will contain one and only one element for each possible row vector value.

D.1.3 Sum of rows vectors that maps to the quotient set

For a given $a \neq I$, the number of elements in G that commute with a will always equal to the number of elements that anti-commute with a. Hence, we have

$$\sum_{g \in G} \zeta(g, a) = 0 \quad \forall a \in A \text{ and } a \neq I$$

As we mentioned before, all $g \in G$ that are within the same coset will have the same row vector value $\zeta(g, a_j)$. Hence, the sum over $\sum_{g \in G}$ can be divided into the sum within the same coset, which is a sum over $|K_A|$ identical row vectors, and the sum over all different cosets. Hence, for all $a \neq I$, we have

$$\begin{aligned} \sum_{g \in G} \zeta(g, a) &= 0 \\ |K_A| \sum_{q_a \in Q_A} \zeta(q_a, a) &= 0 \\ \sum_{q_a \in Q_A} \zeta(q_a, a) &= 0 \end{aligned}$$

i.e.

$$\sum_{q_a \in Q_A} \zeta(q_a, a) = 0 \quad \forall\, a \in A \text{ and } a \neq I \tag{S4}$$

D.2 Quotient Table $\zeta(q_i, h_j)$

D.2.1 Composing the rows of the generator tables

The definition of a generator table $\zeta(\widetilde{q}_i, \widetilde{h}_j)$ is laid out in Section 4.1.2.

We will define Q to be the full set of elements that can be generated from $\widetilde{Q}$:

$$Q = \left\langle \widetilde{Q} \right\rangle$$

Just like how we can generate $q \in Q$ using $\widetilde{q} \in \widetilde{Q}$, we can generate new rows using (9):

$$q = \widetilde{q} * \widetilde{q}' \mapsto \zeta(q, \widetilde{h}_j) = \zeta(\widetilde{q} * \widetilde{q}', \widetilde{h}_j) = \zeta(\widetilde{q}, \widetilde{h}_j)\zeta(\widetilde{q}', \widetilde{h}_j)$$

By composing rows in Table.1 in every possible way, we obtain the new commutator tables $\zeta(q_i, \widetilde{h}_j)$ for different $\left|\widetilde{H}\right|$ in Table.1.

We can see the rows of $\zeta(q_i, \widetilde{h}_j)$ consist of all possible values of $(\pm 1, \pm 1 \cdots)$ vectors of length $\left|\widetilde{H}\right|$. Looking back at the definition of quotient sets in Section.D.1.2, we realise that Q **is just the quotient set of** $\widetilde{H}$.

	$\widetilde{h}_1$
q_1	1
q_2	-1

$\left|\widetilde{H}\right| = 1$

	$\widetilde{h}_1$	$\widetilde{h}_2$
q_1	1	1
q_2	1	-1
q_3	-1	1
q_4	-1	-1

$\left|\widetilde{H}\right| = 2$

	$\widetilde{h}_1$	$\widetilde{h}_2$	$\widetilde{h}_3$
q_1	1	1	1
q_2	1	1	-1
q_3	1	-1	1
q_4	1	-1	-1
q_5	-1	1	1
q_6	-1	1	-1
q_7	-1	-1	1
q_8	-1	-1	-1

$\left|\widetilde{H}\right| = 3$

$\cdots\cdots$

Table S1. Commutator table $\zeta(q_i, \widetilde{h}_j)$ for different $\left|\widetilde{H}\right|$

D.2.2 Composing the columns of $\zeta(q_i, \widetilde{h}_j)$

We will define H to be the full set of elements that can be generated from $\widetilde{H}$:

$$H = \left\langle \widetilde{H} \right\rangle \tag{S5}$$

Just like how we can generate $h \in H$ using $\widetilde{h} \in \widetilde{H}$, we can generate new columns using (9):

$$h = \widetilde{h} * \widetilde{h}' \mapsto \zeta(q_i, h) = \zeta(q_i, \widetilde{h} * \widetilde{h}') = \zeta(q_i, \widetilde{h})\zeta(q_i, \widetilde{h}')$$

If the row $\zeta(g, \widetilde{h}_j) = (1, 1, \cdots, 1)$, then the row $\zeta(g, h_j) = (1, 1, \cdots, 1)$. Hence, H and $\widetilde{H}$ share the same kernel subgroup (see Section.D.1), and thus the same quotient set Q.

By composing all possible columns of the commutator tables in Table.1, we obtain Table.2.

	I	$\widetilde{h}_1$
q_1	1	1
q_2	1	-1

$\left|\widetilde{H}\right| = 1$

	I	$\widetilde{h}_1$	$\widetilde{h}_2$	$\widetilde{h}_1 * \widetilde{h}_2$
q_1	1	1	1	1
q_2	1	1	-1	-1
q_3	1	-1	1	-1
q_4	1	-1	-1	1

$\left|\widetilde{H}\right| = 2$

	I	$\widetilde{h}_1$	$\widetilde{h}_2$	$\widetilde{h}_3$	$\widetilde{h}_1 * \widetilde{h}_2$	$\widetilde{h}_1 * \widetilde{h}_3$	$\widetilde{h}_2 * \widetilde{h}_3$	$\widetilde{h}_1 * \widetilde{h}_2 * \widetilde{h}_3$
q_1	1	1	1	1	1	1	1	1
q_2	1	1	1	-1	1	-1	-1	-1
q_3	1	1	-1	1	-1	1	-1	-1
q_4	1	1	-1	-1	-1	-1	1	1
q_5	1	-1	1	1	-1	-1	1	-1
q_6	1	-1	1	-1	-1	1	-1	1
q_7	1	-1	-1	1	1	-1	-1	1
q_8	1	-1	-1	-1	1	1	1	-1

$\left|\widetilde{H}\right| = 3$

Table S2. Quotient table $\zeta(q_i, h_j)$ for different $\left|\widetilde{H}\right|$

Due to the symmetry between $\widetilde{Q}$ and $\widetilde{H}$, we know that H is also the quotient set of $\widetilde{Q}$. Hence, $\zeta(q_i, h_j)$ is called a quotient table.

D.2.3 Shape of quotient tables

Any $h \in H$ that can be generated from $\widetilde{h}_i \in \widetilde{H}$ can be written as

$$h = \prod_{i=1}^{|\widetilde{H}|} \widetilde{h}_i^{\alpha_i}$$

where the $\prod$ and the exponentials here are all defined in terms of operation $*$.

$\widetilde{h}_i * \widetilde{h}_i = I$ for all $\widetilde{h}_i \in \widetilde{H} \subseteq G$, thus α_i is either 0 or 1. Hence, there are $2^{|\widetilde{H}|}$ possible choice for $\{\alpha_i\}$, which means

$$|H| = 2^{|\widetilde{H}|}$$

Similarly

$$|Q| = 2^{|\widetilde{Q}|} = 2^{|\widetilde{H}|}$$

where we have used (10).

Hence, for a quotient table

$$\begin{aligned} \text{No. of rows} &= |Q| = 2^{|\widetilde{H}|} \\ \text{No. of columns} &= |H| = 2^{|\widetilde{H}|} \end{aligned} \tag{S6}$$

D.2.4 Sum of rows in a quotient table

From (S4), with $A = H$, we have

$$\sum_{q \in Q} \zeta(q, h) = 0 \quad \forall\, h \in H \text{ and } h \neq I \tag{S7}$$

When we compose any two different elements in H, we will just get another non-identity elements in H:

$$h_i = h_j * h_k \qquad \text{where } h_i, h_j, h_k \in H,\ h_j \neq h_k,\ h_i \neq I$$

Substituting into (S7), we have

$$\sum_{q \in Q} \zeta(q, h_j * h_k) = 0 \quad \forall\, h_j,\ h_k \in H \text{ and } h_j \neq h_k \tag{S8}$$

E Constructing the Twirling Set W

E.1 Another View on Requirements of Twirling Set

A noise channel can be decomposed into its Pauli basis V.

In this notation, to fully twirl the noise, we need the twirling set W to satisfy the following equation (using (7) and (2)):

$$\sum_{w \in W} \zeta(w, v * v') = 0 \quad \forall v, v' \in V \text{ and } v \neq v' \tag{S9}$$

which is just (S8) with the following **bijective** mappings:

$$\begin{aligned} W &\mapsto Q \\ V &\mapsto H_V \subseteq H \end{aligned}$$

which can be simplified to

$$\begin{aligned} \widetilde{W} &\mapsto Q_{\widetilde{W}} \subseteq Q \\ \widetilde{V} &\mapsto H_{\widetilde{V}} \subseteq H. \end{aligned}$$

Remember that $Q = \left\langle \widetilde{Q} \right\rangle$. If we want to find the **smallest** W that maps to Q, the only way is to have $Q_{\widetilde{W}} = \widetilde{Q}$ and $W = \left\langle \widetilde{W} \right\rangle$. Hence, our requirement on the twirling set becomes finding the following mappings

$$\begin{aligned} \widetilde{W} &\mapsto \widetilde{Q} \\ \widetilde{V} &\mapsto H_{\widetilde{V}} \subseteq H. \end{aligned} \tag{S10}$$

The way to find such mapping is outlined in Section 4.2.

Looking back at the definition of one-gate twirling in Section 3.3, we realise that doing twirling with W is equivalent to doing nested one-gate twirling with the elements in $\widetilde{W}$:

$$\prod_{\widetilde{w} \in \widetilde{W}} \mathscr{T}_{\{I, \widetilde{w}\}} = \mathscr{T}_W.$$

Hence, after finding the $\widetilde{W}$ that satisfies the mapping in (S10), we can fully twirl the given error M by doing nested one-gate twirling with all the elements in $\widetilde{W}$.

E.2 Size Matching

To achieve the mapping in (S10), we need to ensure the sizes of the sets match each other:

$$\begin{aligned} \left|\widetilde{W}\right| &= \left|\widetilde{Q}\right| = \left|\widetilde{H}\right| \\ |V| &= |H_V| \leq |H| = 2^{\left|\widetilde{H}\right|} \end{aligned} \tag{S11}$$

where we have used (10) and (S6).

From the first equation, we know that minimising $\left|\widetilde{W}\right|$ is the same as minimising $\left|\widetilde{H}\right|$.

From the second equation, we know that

$$|V| \leq 2^{\left|\widetilde{H}\right|} \tag{S12}$$

Since we are looking for the **smallest** $\widetilde{H}$ that satisfy (S12) and $|V| \leq 2^{\left|\widetilde{V}\right|}$, we have:

$$\left|\widetilde{H}\right| \leq \left|\widetilde{V}\right|. \tag{S13}$$

F Notation and Definition

- $\overleftarrow{\ }$: Super-operators. e.g. $\overleftarrow{A}\rho = A\rho A^{\dagger}$.
- $*$: See Section 2.1.
- ζ: See Section 2.2.
- $\widetilde{\ }$: Generating set. Note that $\widetilde{A}$ means that A can be generated from $\widetilde{A}$, but does not means that A is the **complete** set of elements that can be generated from $\widetilde{A}$.
- $\langle\ \rangle$: The full set of operators that can be generated from the given set. In the context of our paper, the composition rules used in the generation is $*$.
- G: The Pauli operator set.
- M: The noise operator that we want to twirl.
- V: The Pauli basis of the noise M.
- W: The twirling gate set.
- Q: The set of Pauli operators that denote the rows of quotient tables.
- H: The set of Pauli operators that denote the columns of quotient tables.
